# The association among ball speed and the rotation of pivot leg, pelvis, and trunk separation in collegiate baseball pitchers

**DOI:** 10.1016/j.heliyon.2025.e42314

**Published:** 2025-01-28

**Authors:** Naoki Wada, Mitsuo Otsuka, Yuta Yamaguchi, Takehiko Tsuji, Takatoshi Kojo, Tokuyoshi Kono, Tetsunari Nishiyama

**Affiliations:** a Faculty of Sport Science, Nippon Sport Science University, 1221-1 Kamoshida-cho, Aoba-ku, Yokohama-shi, Kanagawa, Japan; b Faculty of Sport Management, Nippon Sport Science University, 1221-1 Kamoshida-cho, Aoba-ku, Yokohama-shi, Kanagawa, Japan; c Faculty of Medical Science, Nippon Sport Science University, 1221-1 Kamoshida-cho, Aoba-ku, Yokohama-shi, Kanagawa, Japan

**Keywords:** Biomechanics, Pitching, Lower limb, Torso, Ball speed

## Abstract

In baseball pitching, pivot leg is known to play an important role in linear acceleration down the mound. However, its contribution to the rotational movements of the proximal segments such as pelvis and upper body remains unknown. This study aimed to investigate the association between the pivot leg rotation, pelvis rotation, and trunk separation during the stride phase of baseball pitching in relation to ball speed. Three-dimensional kinematic data from 18 collegiate baseball pitchers were obtained during the maximal-effort pitches. The rotational angles and angular velocities of the pivot leg, pelvis, and upper trunk in the stride phase of pitching delivery were calculated to investigate the association of pivot leg rotation with pelvis rotation, upper trunk rotation, and trunk separation. The results showed that the rotational angular displacement of the pivot leg was positively correlated with pelvis rotation and negatively correlated with trunk separation during the stride phase and at the instant of stride foot contact. The peak rotation angular velocity of the pivot leg was also negatively correlated with trunk separation angle at stride foot contact. The trunk separation angle at the stride foot contact was the only variable that was positively correlated with ball speed. These results indicated that the pivot leg and pelvis behave similarly in terms of rotational movement in the transverse plane. Achieving greater trunk separation at stride foot contact, mainly by increasing pelvis rotation in association with pivot leg rotation, might increase ball speed. These findings can be useful when the coaches give instructions to modify the rotational mechanics of their pitchers.

## 1 Introduction

In baseball, the pitching motion requires complex and proper sequencing of the body segments. During pitching, the ability to transfer momentum up to the throwing arm through kinetic chain by increasing the rotation of the trunk segments allows the pitchers to throw with higher ball speeds while minimizing the potential risk of injury, which was observed in elite pitchers [1]. Previous studies have reported that the pelvis rotation angle and angular velocity in the transverse plane were positively correlated with ball speeds [[2], [3], [4]] and throwing-arm linear and rotational kinetics [5]. Near the instant of stride foot contact in particular, pelvis rotation in a greater amount toward home plate followed by delayed upper trunk rotation creates the rotational lag [6]. This enhances the effect of stretch-shortening cycle in the core musculatures to produce larger and faster rotation of the trunk and consequently an acceleration of the throwing arm and a ball [7].

Functionally, pelvis rotation can be driven by internal and external rotation of the legs in the transverse plane. When a right-handed pitcher begins to rotate the pelvis internally before stride foot contact, the right thigh, as a pivot leg, and the left thigh, as a stride leg, rotate in the same direction. The investigation of the hip motion in relation to the spine reported that the right hip medial rotation was greater when twisting the trunk rightward compared to the left hip lateral rotation [8]. This indicates that, for the right-handed pitcher, an internal rotation of the pivot leg plays an important role in rotating the upper trunk clockwise over the pelvis to achieve greater trunk separation, which is referred to as the “X-factor” of the pitching [9]. Although the stride leg behavior is also believed to have a direct influence on the pelvis rotation, altering the stride leg kinematics during stride phase might affect the landing position or the direction of the stride foot which could decrease the consistency in pitch location [10]. Therefore, it would be ideal to rather modify the mechanics of pivot leg than that of stride leg when attempting to change the rotational mechanics of the pelvis and trunk in the transverse plane.

Some previous studies included the hip internal and external rotations as kinematic parameters to investigate their relationships with performance or injury indicators [8,[11], [12], [13], [14]]. One study reported that the role of back (pivot) leg changes from generating forward linear momentum to angular momentum during pitching [14]. This indicates that the pivot leg may contribute not only to the translational acceleration down the pitcher's mound by generating a push-off force against the rubber plate but also to the transition into rotational movement. Another study reported that the pivot leg external rotation torque was the main factor that rotates pelvis [15]. However, due to the anatomical structure, prolonged external rotation during the stride phase is assumed to inhibit the pelvis to further increase its rotation toward home plate. Thus, the pivot leg may need to rotate along with the pelvis to help those two segments align properly. While the pivot leg and pelvis in the transverse plane are assumed to rotate in the similar manner, no previous study has reported the coordinated movement of those two segments. Therefore, the purpose of this study was to investigate the association between the rotational movement of the pivot leg, pelvis, and upper trunk during the stride phase. It was hypothesized that pitchers with greater internal rotation of the pivot leg would show greater pelvis rotation and trunk separation during the stride phase.

## 2 Methods

### 2.1 Participants

After recruiting participants for this study two weeks prior to data collection, 18 male collegiate pitchers (age: 20.1 ± 1.0 years old, height: 1.77 ± 0.06 m, body mass: 77.4 ± 8.0 kg) participated in this experiment, including 13 right-handed and 5 left-handed pitchers. All participants were informed of the purpose, procedure, and data handling and provided written consent to participate in this study. The participants were then asked to complete a questionnaire regarding their anthropometrics, medical history, and injury history. Inclusion criteria for the participation of this study included 1) no injury history of trunk and upper or lower extremity within a year prior to the measurement, and 2) at least 3 years of experience in pitching primarily as a pitcher. None of the participants were excluded from this study due to the inability of participation since they all have met the criteria. This study was conducted in accordance with the guidelines of the Declaration of Helsinki and approved by Nippon Sport Science University Board of Ethics (021-H109).

### 2.2 Data collection

**Fig. 1:**
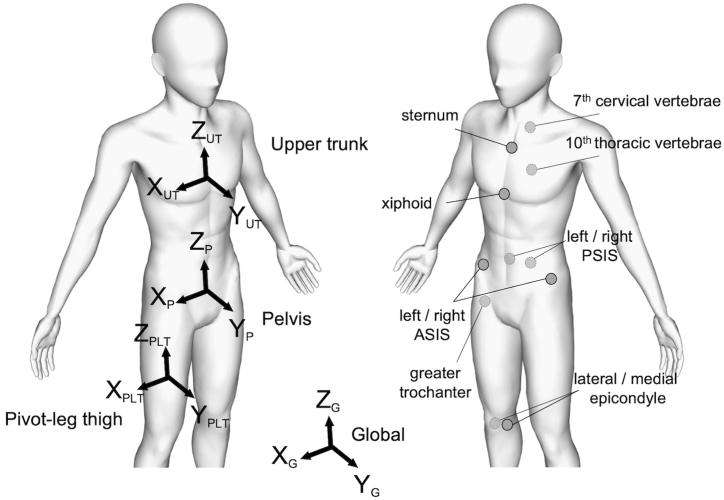
Marker settings and coordinate systems for pivot leg, pelvis, and upper trunk segments.

Sixty-two reflective markers were placed on the anatomical landmarks of the body segments, in which 11 of those markers were used to identify the pivot leg thigh, upper trunk, and pelvis segments of each participant (Fig. 1). Three-dimensional motion capture system with 12 cameras (Arqus 5; Qualisys Inc., Gothenburg, Sweden) captured the position of each reflective marker at 250 Hz. Ball speed was measured using a Speed Gun (1GJYM20100; MIZUNO, Osaka, Japan) placed 3 m behind and in line with the catcher. After a sufficient self-preferred warm up, the participants were instructed to throw ten maximum effort pitches from the stretch position aimed at the catcher, 18.44 m away from the pitcher's mound in the indoor practice field. The trials were considered to be successful when the balls were caught within the range of strike zone judged by the catcher and one of the conductors of this study. The pitches were recorded as failure if 1) the balls were caught out of the strike zone or 2) the coordinate data was unable to be collected due to the detachment of the reflective markers on the participant's body segments.

### 2.3 Data analysis

The global coordinate system was defined with the axes in X, Y, and Z directions (X_G_, Y_G_, and Z_G_, respectively). X_G_ and Y_G_ were the unit vectors pointing in the third base direction and throwing direction, respectively. Z_G_ was the cross-product of Y_G_ and X_G_ pointing in the upward direction (Fig. 1). The time-series marker position data were filtered in all X, Y, and Z directions independently with 16.7 Hz fourth-order Butterworth low-pass filter, as suggested by previous studies [16,17].

The pivot leg thigh was identified from the markers placed on the greater trochanter and medial and lateral epicondyles of the femur bones. For the Z-axis of pivot leg thigh (Z_PLT_) to be defined, right hip joint center was estimated based on the anteroposterior and mediolateral lengths of the pelvis segment [18]. Z_PLT_ was then calculated as the unit vector in the direction from the midpoint of left and right femoral epicondyles to right hip joint center. The Y-axis of pivot leg thigh (Y_PLT_) was the cross-product of the unit vector in the direction from medial to lateral epicondyle and Z_PLT_. The X-axis of pivot leg thigh (X_PLT_) was the cross-product of Z_PLT_ and Y_PLT_. The pelvis and upper trunk segments were identified from the markers placed on the right and left anterior superior iliac spines, right and left posterior superior iliac spines, seventh cervical vertebra, tenth thoracic vertebra, upper edge of the sternum, and lower end of the xiphoid process. The segmental coordinate systems of upper trunk and pelvis were established based on the International Society of Biomechanics recommendations except X-axis directed laterally from left to right, Y-axis directed anteriorly, and Z-axis directed superiorly [[19], [20], [21]]. The unit vector pointing from left to right was defined as the X-axis of pelvis (X_P_) and upper trunk (X_UT_). The Z-axis of pelvis (Z_P_) and upper trunk (Z_UT_) were the unit vectors pointing upward. The Y-axis of pelvis (Y_P_) and upper trunk (Y_UT_) were the cross-products of X_P_ and Z_P_, and X_UT_ and Z_UT_, respectively (Fig. 1).

The rotational angles of pivot leg thigh, pelvis, and upper trunk segment in the transverse plane formed between the global coordinate system and each segment's coordinate system were calculated using MATLAB software (R2021a; MathWorks, Massachusetts, USA). Because the kinematic differences between left-handed and right-handed pitchers were reported to have a minimal practical significance [22], the coordinate data of left-handed pitchers were converted to right-handed data before calculating angles and angular velocities for the comparison purpose. The pivot leg thigh, pelvis, and upper trunk rotation angles were defined as 0° when the anteroposterior axes of the segments' coordinate system (Y_PLT_, Y_P_, and Y_UT_) were parallel to Y_G_, and 90° when they were parallel to X_G_. Trunk separation angle was defined as the upper trunk rotation angle minus pelvis rotation angle in the transverse plane, and the value was positive when upper trunk was rotated clockwise relative to pelvis. The segmental angular velocity was calculated as the derivative of the segmental angle with respect to time.

The pitching cycle was defined as the normalized interval between two instants during the pitching delivery, from maximum knee elevation (0 %) to ball release (100 %). The stride phase was defined as the interval between maximum knee elevation and stride foot contact. Maximum knee elevation was the instant when the knee joint center of stride leg achieved the maximal vertical height, and stride foot contact was identified as the instant when the ankle marker of the stride leg first decreased its velocity to less than 1.5 m/s [4,16,18,23]. Ball release was defined as the instant 0.01 s after the wrist joint center of throwing arm passed that of elbow joint center in the Y_G_ direction [9,18,23,24].

### 2.4 Statistical analysis

For each participant, the three pitches with the highest ball speeds were used to calculate the mean and standard deviation of the kinematic variables during the stride phase and at the instant of stride foot contact. The peak angular velocity of the pivot leg thigh was calculated as the peak value observed during the stride phase, whereas those of the pelvis and upper trunk were calculated as the peak values observed throughout the pitching cycle. Spearman's rank correlation coefficient (ρ) was calculated for the relationship among the pivot leg rotation, pelvis rotation, trunk separation, and ball speed. The correlation coefficient was interpreted as weak (<0.3), moderate (0.3–0.5), or strong (>0.5) per the recommendation of previous studies [25,26]. Statistical significance was set at p < 0.05. All the calculations were performed using MATLAB software (R2021a; MathWorks, Massachusetts, USA).

## 3 Results

**Fig. 2:**
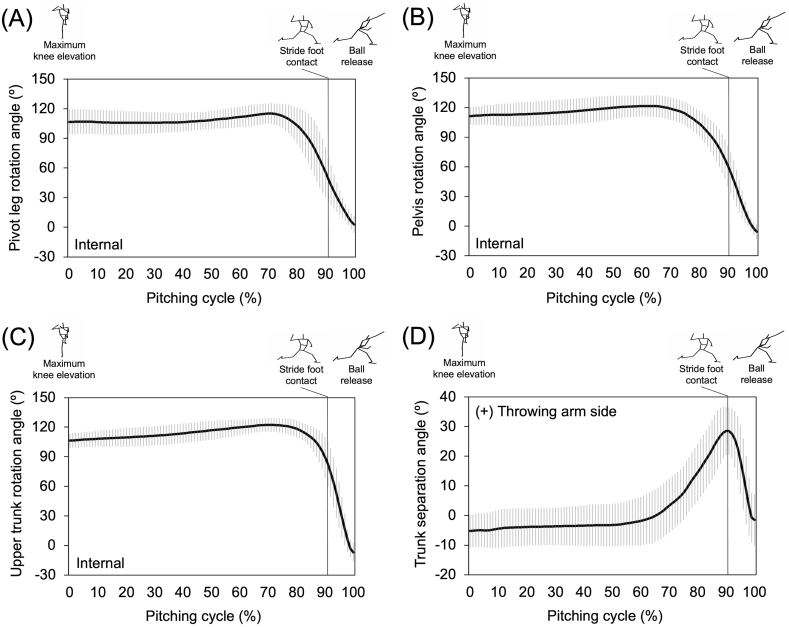
Mean and standard deviation of time-series pivot leg rotation, pelvis rotation, and trunk separation angles of all participants. (A) Pivot leg rotation (B) Pelvis rotation (C) Upper trunk rotation (D) Trunk separation. The data were normalized with respect to time during the pitching cycle (0 %: maximum stride leg knee elevation, 100 %: ball release).

**Fig. 3:**
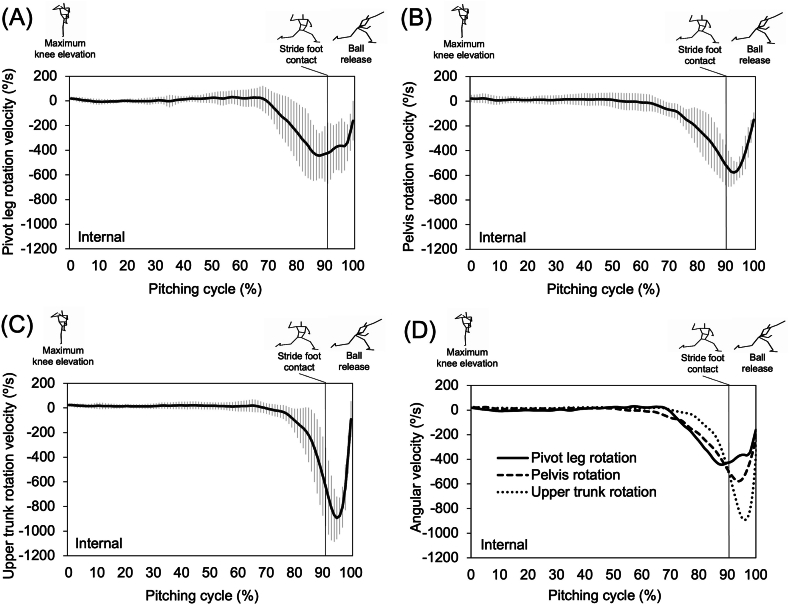
Mean and standard deviation of time-series pivot leg, pelvis, and upper trunk rotation angular velocities of all participants. (A) Pivot leg rotation (B) Pelvis rotation (C) Upper trunk rotation (D) Overlapped pivot leg, pelvis, and upper trunk rotation. The data were normalized with respect to time during the pitching cycle (0 %: maximum stride leg knee elevation, 100 %: ball release).

The mean ball speed was 134.2 ± 5.7 km/h. Pivot leg, pelvis, and upper trunk began to increase the internal rotation before the instant of stride foot contact, and it continued increasing until ball release. At stride foot contact, the internal rotation angle of the pivot leg was the greatest, followed by pelvis and upper trunk, respectively (Fig. 2 (A–C)). The trunk separation angle peaked near stride foot contact and decreased toward ball release as the upper trunk rotation caught up with the pelvis rotation (Fig. 2 (D)). The pivot leg internal rotation angular velocity reached its peak before stride foot contact and then decreased until ball release, whereas pelvis and upper trunk rotation angular velocities peaked after stride foot contact (Fig. 3 (A–C)). Peak internal rotation angular velocities of the pivot leg, pelvis, and upper trunk were observed in sequence (Fig. 3 (D)).

The means and standard deviations (SD) of the kinematic variables are listed in Table 1. Pivot leg rotation angular displacement during the stride phase was positively correlated with pelvis rotation angular displacement during the stride phase (ρ = 0.70, p < 0.01) and pelvis rotation angle at stride foot contact (ρ = 0.64, p < 0.01). In addition, it was negatively correlated with trunk separation angular displacement during the stride phase (ρ = −0.56, p < 0.05) and trunk separation angle at stride foot contact (ρ = −0.49, p < 0.05). Peak rotation angular velocity of the pivot leg during the stride phase was negatively correlated with trunk separation angle at stride foot contact (ρ = −0.57, p < 0.05). The trunk separation angle at the instant of stride foot contact was positively correlated with ball speed (ρ = 0.50, p < 0.05).

**Table 1:** Mean and standard deviation (SD) of pivot leg, pelvis, and trunk rotational kinematics.

Phase/Instant	Variable	Mean ± SD
Stride phase	Pivot leg rotation angular displacement (°)	–70 ± 15
	Pelvis rotation angular displacement (°)	–68 ± 14
	Upper trunk rotation angular displacement (°)	–32 ± 14
	Trunk separation angular displacement (°)	36 ± 8
Stride foot contact	Pivot leg rotation angle (°)	37 ± 12
	Pelvis rotation angle (°)	43 ± 8
	Upper trunk rotation angle (°)	74 ± 9
	Trunk separation angle (°)	31 ± 7
Ocurrence of timing of peak value	Pivot leg rotation angular velocity (°/s)	–732 ± 129
	Pelvis rotation angular velocity (°/s)	–659 ± 63
	Upper trunk rotation angular velocity (°/s)	–1025 ± 124

Pivot leg, pelvis, and upper trunk rotation angle: (0°) home plate direction, (90°) third base direction. Angular displacement and velocity: (−) internal rotation, (+) external rotation. Trunk separation: upper trunk (−) counterclockwise, (+) clockwise rotation relative to pelvis.

## 4 Discussion

The purpose of this study was to examine how internal rotation of the pivot leg is related to pelvis rotation and trunk separation during the stride phase. The pivot leg rotation showed strong correlations between pelvis rotation and trunk separation during the stride phase and at stride foot contact, which supported the hypothesis. The results of this study demonstrate that the pitchers may be able to achieve greater pelvis rotation and trunk separation when the pivot leg rotation also increases before stride foot contact, which potentially contributes to an increase in ball speed.

In the latter half of the pitching cycle where the linear forward movement almost completes and transitions into the rotational movement [27], the pelvis needs to rotate toward the home plate prior to the upper trunk for the proper sequencing of the segments [28]. In fact, a previous study having 157 professional pitchers divided into open- and closed-pelvis groups found that the pitchers with greater pelvis rotation angle at stride foot contact had higher ball velocity [2]. Another previous study on the pelvis rotation styles reported that the pitchers with early rotation (greater pelvis rotation at stride foot contact) showed lower kinetic values for their shoulders and elbows [5]. These findings indicate that increasing pelvis rotation during the stride phase and achieving greater angle by the time of stride foot contact would be beneficial for the pitchers to both enhance the performance and reduce the stress on the throwing arm. For the pelvis to rotate during the stride phase, it is assumed that the pivot leg also needs to rotate internally along with the pelvis for the functionally proper alignment between the acetabulum and femoral head at the hip joint [29]. The results of this study indicate that the internal rotation of pivot leg thigh segment might assist proper rotation of the pelvis to produce higher ball speed, supported by a previous study reporting that the increase in the angular velocity of the thigh in the pivot leg led to an increase of the pelvis angular velocity [15].

In addition, the trunk separation angle at stride foot contact was also correlated with ball speed. The rotational lag between pelvis and upper trunk causes the stretching of abdominal muscles and tendons with stored elastic energy. Due to the stretch-shortening cycle, this energy is then released and does the work of enhancing the upper trunk rotation, referred to as the “serape effect” [9]. Similarly, a previous study on 30 elite pitchers found that the proper mechanics in producing higher ball speeds involved greater trunk separation with greater pelvis rotation at stride foot contact [3]. Another previous study reported that the upper trunk rotation angle (defined as trunk separation angle in this study) was greater in professional pitchers with higher ball velocity but lesser normalized elbow varus torque compared to high school pitchers [4]. Those reports suggest that the pitchers should be able to achieve greater trunk separation by the time of stride foot contact for enhancing the performance while reducing the risk of arm injuries. In this study, pitchers with greater trunk separation showed greater and faster rotation of the pivot leg thigh segment. Because the pivot leg thigh segment is linked only to the pelvis segment at its proximal end and may not affect directly to the upper trunk segment, it is possible that the pivot leg rotation helps increasing the rotational difference between pelvis and upper trunk in the transverse plane. Since arm kinetics was not the interest of this study, further research is warranted to investigate the role of pivot leg rotation in producing higher ball speeds while minimizing the throwing arm stress.

The previous studies found that there was a positive correlation between trunk separation angle at stride foot contact and peak upper trunk rotation velocity which was directly or indirectly associated with ball velocity [7,30], indicating that the faster rotation of upper trunk contributed to accelerating the distal end of the kinetic chain. Although no peak rotation angular velocity of the segments showed correlations with ball speeds in this study, the peak angular velocity of pivot leg is believed to play an important role in transferring the mechanical energy to the throwing arm and producing ball speeds [15,29]. The peak angular velocities were observed in order from proximal to distal (Fig. 3 (D)). The lag between those peak segment velocities seems to influence the production of ball speed, as the previous study reported that the pitchers with higher ball speeds showed lesser upper trunk rotation at the time of peak pelvis rotation velocity [31]. Therefore, higher rotational velocity of the pivot leg might consequently increase the upper trunk rotational velocity through pelvis, the concept known as the summation of speed principle [32], reported in other previous studies on rotational sport movements [33,34]. The previous findings and the results of this study together indicate that the greater and faster rotation of the pivot leg may play an important role in assisting the pelvis rotation while preventing the early opening of the upper trunk in order to achieve a highly accelerated and properly timed upper trunk rotation.

Some previous studies on pitching kinematics included a variable referred to as hip internal rotation, the thigh segment angle relative to the pelvis in the transverse plane, which was similar to the pivot leg rotation in this study. The hip internal rotation of the pivot leg at the instant of maximum knee height and hand separation (both occur during the stride phase of this study) was reported to be greater in the professional pitchers than high school pitchers [1]. Similarly, another previous study reported that whereas the kinematics of the pivot leg during the pitching cycle was not significantly correlated with ball speed, the hip internal rotation torque of the pivot leg at the timing of maximal anterior push-off force (occurs during the stride phase of this study) was significantly greater in the pitchers with higher ball speeds [11]. Regarding the hip rotation and throwing arm kinetics, the hip internal rotation of the trailing (pivot) leg at stride foot contact did not have a significant correlation with peak elbow varus torque [35]. Although the hip internal rotation is a variable relative to pelvis unlike the independent rotation of the pivot leg segment relative to global coordinate system in this study, these findings imply that maintaining the hip internal rotation range of motion of the pivot leg could prevent overloading of the throwing arm [15]. Because hip internal rotation is, by definition, highly dependent on the orientation of pelvis, the pivot leg rotation examined in this study was intended to represent the rotation of pivot leg thigh that is isolated from other segments. Therefore, greater angular displacement of the pivot leg rotation during the stride phase may be important and applicable to any pitcher with insufficient pelvis rotation or trunk separation.

Regardless of the intent to execute a certain pitching motion, the pivot leg kinematics can be limited by segmental mobility and flexibility. Previous study reported that the passive range of motion of the pivot leg hip rotation was correlated with pelvis rotation angle at stride foot contact [36]. Another study found that, although no correlation between pivot leg range of motion and any pitching kinematics or ball velocity were observed, the pitchers with greater internal rotation range of motion of the stance (pivot) leg over lead (stride) leg showed greater odds of displaying the trunk separation above the recommended amount [30]. In addition, the collegiate pitchers with greater trail (pivot) leg hip range of motion showed the later onset of maximum trunk rotational angular velocity in the pitching cycle [37], which is referred to as proper sequencing of the segments. From the clinical perspective, the internal rotation range of motion of the trail (pivot) leg with 90° hip flexion was greater in healthy adolescent pitchers than in their counterparts with elbow pain [38]. These findings, along with the results of this study, suggest that producing greater and faster internal rotation of the pivot leg is an important mechanics in baseball pitching that should be stressed by coaches. This instruction would be highly effective when the pitchers attempt to rotate their pelvis more toward the home plate and achieve greater trunk separation during the stride phase. It should also be ensured that there is no lack of functional mobility and flexibility at the hip joint before modifying the pivot leg mechanics.

The limitation of this study was that only the overhand pitchers participated, and the sidearm or underhand pitchers were not included even though the different pitching styles have different kinematics [24]. Pelvis rotation and trunk separation in the transverse plane were reported to be significantly different among overhand, three-quarter, and sidearm pitching styles [39]. This implies that pitchers with styles other than overhand might show different patterns of trunk, pelvis, or pivot leg kinematics and correlational relationships among those segments. In addition, this study only recruited male collegiate pitchers and did not include youth, high school, or female pitchers. Future studies considering various age, gender, level of competition, and pitching styles are warranted to ensure that the findings of this study can be used to a larger population of pitchers.

## 5 Conclusion

This study investigated the kinematics of pivot leg rotation during the stride phase and its correlation with pelvis rotation and trunk separation. The results showed that the pitchers with greater pivot leg rotation and its peak angular velocity achieved greater pelvis rotation and trunk separation during the stride phase and at the instant of stride foot contact. Because trunk separation was correlated with ball speed in this study, rotating the pivot leg more toward the home plate before the leading foot contacts the ground may have helped increasing the pelvis rotation and thus trunk separation, which is suggested to be the proper mechanics of pitching that produces higher ball speed.

## CRediT authorship contribution statement

**Naoki Wada:** Writing – review & editing, Writing – original draft, Visualization, Validation, Software, Resources, Project administration, Methodology, Investigation, Formal analysis, Data curation, Conceptualization. **Mitsuo Otsuka:** Writing – review & editing, Validation, Supervision, Software, Methodology. **Yuta Yamaguchi:** Writing – review & editing, Software, Methodology. **Takehiko Tsuji:** Writing – review & editing, Resources, Conceptualization. **Takatoshi Kojo:** Writing – review & editing, Resources. **Tokuyoshi Kono:** Writing – review & editing, Resources, Conceptualization. **Tetsunari Nishiyama:** Writing – review & editing, Supervision, Resources, Project administration.

## Consent to participate

All participants were informed of the purpose, procedure, and data handling and provided written consent to participate in this study.

## Ethical considerations

This study was conducted in accordance with the guidelines of the Declaration of Helsinki and approved by Nippon Sport Science University Board of Ethics (021-H109).

## Consent for publication

The written informed consent including the consent for publication, along with the consent to participate, was obtained from all participants.

## Data availability

The datasets generated and/or analyzed during the current study are not publicly available but are available from the first author Naoki Wada on reasonable request.

## Funding

The authors have received no financial support for the research, authorship, or publication of this article.

## Declaration of competing interest

The authors declare no conflicts of interest associated with this manuscript.
